# Group intellectual transparency: a novel case for non-summativism

**DOI:** 10.1007/s11229-022-03617-x

**Published:** 2022-03-05

**Authors:** T. Ryan Byerly

**Affiliations:** Department of Philosophy, University of Sheffield, 45 Victoria St, Sheffield, S3 7QB UK

**Keywords:** Transparency, Intellectual virtue, Collective epistemology, Perspective, Summativism

## Abstract

Philosophical reflection on transparency, including group transparency, is beginning to gain steam. This paper contributes to this work by developing a conceptualization of transparency as an intellectual character trait that groups can possess, and by presenting a novel argument for thinking that such transparency should be understood along non-summativist lines. According to the account offered, a group’s being intellectually transparent consists in the group’s tending to attend well to its perspective and to share its perspective faithfully with others in order to promote their epistemic goods. It is argued that this kind of group intellectual transparency, pace summativism, does not always consist merely in group members possessing intellectual transparency. The argument given for this conclusion works differently from existing arguments for non-summativism about group character traits, and it retains persuasive power even if summativist views of most group phenomena, including other group character traits, are correct.

The topic of intellectual transparency has received very limited attention from philosophers. Yet, appeals are increasingly made to transparency, including intellectual transparency, in many contexts of life. This is especially so with respect to academic research, particularly in the sciences (e.g., Elliot, forthcoming), and with respect to organisations’ relationships to their members or employees, stakeholders, and the public (e.g., Kogelmann, forthcoming). As such, it might be valuable for philosophers to devote more attention to the topic. I aim to contribute to these efforts in this paper, focusing on transparency understood as a group intellectual character trait. To treat intellectual transparency in this way, I will draw upon some of my own recent research (2021), which has provided the only philosophical analysis of intellectual transparency as a virtue of individual people that I am aware of, and I will also draw upon the small but rapidly growing literature on group character traits and the broader, maturing literature on group epistemology.

I begin in Sect. 1 by briefly presenting my account of individual intellectual transparency and explaining how it could be adapted to provide an account of group intellectual transparenfcy. I then turn in the longer Sect. 2 to the summativist versus non-summativist debate as applied to intellectual transparency, engaging with the question of whether a group’s possession of intellectual transparency might involve anything more than the group members possessing intellectual transparency as individuals. I will argue for a non-summativist position, doing so in a way that provides a novel route to defending non-summativism. In fact, the argument I will provide arguably works even if simple summativist views of other group intellectual phenomena, including other group intellectual character traits, are correct. The argument thus puts strong pressure on summativism, while identifying some of the distinctive features that may be required for group intellectual transparency beyond the intellectual transparency of individual group members.

## An account of group intellectual transparency

My (2021) approach treats intellectual transparency as an intellectual character trait, conceptualizing it as one of the virtues of the “intellectually dependable person”—the sort of person on whom others can depend in their inquiries. To be this kind of person, I argue, it is not enough to possess true beliefs or knowledge or individual epistemic achievements in general. This is for two reasons. First, a person may have knowledge or true beliefs, but be unwilling or inept at communicating these to others so as to improve others’ inquiries. And, second, we depend on others to do more for us in our inquiries than just to share their knowledge or true beliefs regarding the target propositions of our inquiries: we depend on them to share evidence and ignorance, to raise questions, to evaluate our investigative methods, to model excellent inquiry for us, and so on. Intellectual transparency as I understand it is one of several virtues that helps a person be the sort of person who can be broadly depended upon in these varied ways by others in the pursuit of their inquiries.

Intellectual transparency is concerned specifically with the domain of sharing one’s perspective with others. As a distinctively other-regarding intellectually virtuous character trait, intellectual transparency is oriented toward and ultimately motivated by promoting others’ epistemic goods. Thus, intellectual transparency is “a tendency to faithfully share one’s perspective on topics of others’ inquiries with these others out of a motivation to promote their epistemic goods” (2021, p. 105). The intellectually transparent person values others’ attainment of goods such as knowledge, understanding, and true belief, and they have a tendency to faithfully share their perspective with others so as to promote these kinds of goods. When sharing their perspective can help others attain such goods, they will be *ceteris paribus* inclined to do so; when sharing their perspective wouldn’t help in this way, they won’t be motivated by their transparency to do so. Given the role that a person’s motivations toward epistemic goods play in this account of intellectual transparency, the acocunt is more at home within responsibilist or personalist approaches to intellectual virtue than with reliabilist approaches (cf. Battaly, 2019).

The idea of a “perspective” plays an important role for me, as it does for others (e.g., Riggs, 2019). I understand perspectives to be richly complex things. They include a person’s beliefs, but also their intuitions, experiences, conceptual schemes, arguments, evidential standards, and intellectual tendencies. We might put it this way: when a person shares their perspective with another on the topic of that other’s inquiry, what they share is their “take” on that topic. There’s a lot, beyond just beliefs, that might be a part of such a “take”.

The complexity of perspectives helps to illuminate the role that skill plays in my account of transparency. When I say that the intellectually transparent person tends to “faithfully” share their perspective, I’m alluding to such skill—two kinds of skill, in fact. The first kind of skill pertains to self-knowledge. The intellectually transparent person is good at figuring out what their own perspective in fact is. They’re good at identifying, for instance, whether they believe a claim is false or whether they just don’t believe it is true; and they’re good at identifying what experiences or arguments and evidential standards have played a role in their adopting the views they do adopt toward the claim. Having this sort of self-knowledge isn’t always easy, and in some cases the intellectually transparent person may get it wrong. But part of their intellectual transparency is that they are at least good at getting it right.

Intellectual transparency also involves skills in communicating one’s perspective to others. This requires, for example, a sophisticated vocabulary for distinguishing between such things as when one believes a claim is false and when one does not believe a claim is true, or when one is in possession of an argument for a claim’s truth and when one is in possession of a response to an argument for a claim’s falsity. It also requires skill in enabling others to enter into and appreciate how things appear from one’s perspective. Thus, virtuous intellectual transparency involves skills of self-understanding and skills of self-disclosure to others. As with the skills of self-understanding, the skills of self-disclosure aren’t infallible, and whether others capitalize on what is communicated by the intellectually transparent person is in part up to them.

While there is more that could be said about the ins and outs of intellectual transparency, what has been said thus far should suffice for our purposes. Intellectual transparency, on my recent account, is a character trait of individual people that involves tending to skilfully attend to one’s own perspective and communicate this perspective to others well so as to advance others’ epistemic goods. The focus of the present paper is to consider how this account might be adapted to provide an account of intellectual transparency as a character trait of groups.

It would seem that this adaptation may be accomplished fairly straightforwardly. For a group to have the character trait of intellectually transparency is for the group to tend to skilfully attend to its perspective and to communicate this perspective well to others so as to advance others’ epistemic goods. As in the individual case, such transparency would involve the group in caring about and being motivated to promote others’ epistemic well-being. It will also involve the group in having skills of self-knowledge that enable the group to grasp well the varied aspects of the group’s perspective—the group’s beliefs, evidence, evidential standards, conceptual schemes, intellectual tendencies, and so on. And it will involve skills in communicating these varied aspects of the group’s perspective well to others so as to advance others’ epistemic goods.

I think this basic approach to adapting my account and applying it to the case of groups provides us with a workable understanding of one sort of group-level transparency. Specifically, it provides us with an account of a sort of group-level transparency that is an intellectual character trait, and one that is at least a candidate for being an intellectual virtue of at least some groups. Just as we might imagine and admire individuals who satisfy my account of individual intellectual transparency, we might imagine and admire some groups that satisfy this adapted account of group-level intellectual transparency. For instance, this might be true of research teams. Such teams might tend to skilfully attend to their perspectives and share these effectively with others so as to promote others’ epistemic goods—and we might regard this as a feature that makes them better and more admirable as research teams, contributing to their intellectual worth (cf. Baehr, 2011). On the flip side, as Jennifer Lackey (2020) has emphasized in her recent work, groups may lie, bullshit, or mislead others, or may otherwise refuse to share their perspectives with others or share their perspectives with others only poorly, thereby doing others epistemic harm, and in many cases we may regard such groups with contempt.

Now, it is not my primary aim here to consider whether and for which groups such intellectual transparency would be a virtue. Instead, I am simply suggesting that such intellectual transparency would be a character trait of a group that possessed it, and that it might be a virtue for at least some groups. Briefly, my own view about for which groups such intellectual transparency would be a virtue is that it would depend upon the nature of the groups (see Byerly, forthcoming). Groups that have as one of their important functions being depended upon by others in their inquiries will be better candidates for groups for which such intellectual transparency would be virtuous. Groups that don’t have as one of their important functions being depended upon by others in their inquiries will be less good candidates for groups for which such intellectual transparency would be virtuous. This is because in the former case but not the latter, being intellectually transparent would seem to make the group better as the kind of group that it is, which we might take to be partially definitive of what makes a character trait a virtue for a group.

My main purpose here, however, is not to engage at length with this value question about whether and for which groups intellectual transparency might be virtuous, but rather to engage with a more metaphysically-oriented question about the constitution of group intellectual transparency. While the adapted account of group intellectual transparency presented earlier may seem fine as far as it goes, we might wonder whether such intellectual transparency, when realized by a group, is always ultimately just a matter of group members themselves possessing individual intellectual transparency of the sort identified above, or whether it sometimes involves something further? After all, one might grant that it is fine to talk about groups being intellectually transparent in the sort of way explained by the account given earlier, but contend that for groups to be intellectually transparent in this sense it is necessary and sufficient that their members be individually intellectually transparent. Alternatively, one might contend that for groups to be intellectually transparent in the way explained earlier sometimes involves something beyond the intellectual transparency of individual group members. In the next section, I will be arguing in favor of the latter view. However, before getting to this, I want to conclude this section with two quick clarifying notes about group intellectual transparency as presented thus far.

First, I want to emphasize that I am not suggesting that group intellectual transparency as explained earlier is the only sort of group (intellectual) transparency. As I have suggested, the sort of group transparency identified here is one that is a group intellectual character trait; yet there may be other sorts of group transparency. In addition to talking about transparency as a group intellectual character trait, we may wish to talk about group acts or practices that are or are not (intellectually) transparent. Or we may want to talk about group tendencies to share information that don’t involve the kinds of epistemic motivations characteristic of the kind of intellectual transparency discussed here. Or we might want to talk about norms or activities of requiring (intellectual) transparency of groups, rather than the groups being (intellectually) transparent themselves. Indeed, I think philosophers such as Nguyen (forthcoming) and Kogelmann (forthcoming) have primarily had in mind these alternative kinds of group transparency in their work, and transparency understood in these ways may be the notion that predominates in discourse about transparency in scientific research. Nothing said here should be understood as suggesting that these phenomena are not important topics of investigation or are not appropriately referred to using the language of “transparency”. It is important to keep in mind that we may be referring to different things using similar language.^1^

Second, it seems to me important to point out that there is at least one important disanalogy between an individual’s being intellectually transparent and a group’s being intellectually transparent. Whereas an individual’s being intellectually transparent would not seem to require them to be transparent toward sub-parts of themselves, arguably a group’s being intellectually transparent may require it to be transparent toward subparts of itself. For an individual to be intellectually transparent, they plausibly need only to be intellectually transparent toward others who are not themselves or parts or members of themselves. But for a group to be intellectually transparent, it seems more plausible that they may need to be intellectually transparent toward their members. This may sound a bit odd, but the basic idea is just that there may be cases in which those members of the group (or potentially outside it) who are responsible for attending to and communicating the group’s perspective may need to communicate this perspective to other group members who do not have this responsibility, and not only to outsiders. This point is worth noting in part because the language of transparency is often invoked precisely because of concerns about whether groups are transparent toward their own members or employees. Thus, I would suggest that when we consider the account of intellectual transparency given earlier, we understand the “others” whose epistemic goods the intellectually transparent group is concerned to promote via sharing their perspective to include the group’s own members, in addition to other individuals and groups outside of the group itself.

## A case for non-summativism about group intellectual transparency

In the small but growing literature on group virtues, vices, and character traits,^2^ one of the central philosophical debates is the debate between summativist and non-summativist views. These views are concerned with the relationship between a group’s possession of a virtue, vice, or character trait and group members’ possession of that feature. According to summativist views, as Lahroodi explains,

[A]n ascription of a virtue to a group is always to be understood as a disguised ascription of that virtue to individuals in the group. Thus, to say of a group that it is open-minded is just to say, though indirectly, that its members are open-minded. (411)

And summativist views of vices or character traits would be similar. Whether a group possesses a vice V or a character trait T, according to summativism, is always just a matter of whether its members possess V or T. Summativist views may differ regarding whether all members must possess the relevant feature in order for the group to possess it, or whether only a certain percentage of the members must, or whether only the “operative”, decision-making members of a group or a certain percentage of them must possess the feature in order for the group to possess it. But what is central to summativist views is the contention that a group’s possession of a virtue, vice, or character trait is always just a matter of the right combination of group members possessing that feature.

Non-summativist views reject summativism. They maintain that it is not always the case that a group’s possession of a virtue, vice, or character trait is just a matter of the right combination of the group’s members possessing that feature. To quote Lahroodi again, non-summativism “does not treat collective virtues merely as the sum of the virtues of individual members” (411). And the same holds for non-summativist approaches to vices and character traits. For a group to possess a virtue, vice, or character trait, it sometimes requires more than just the right combination of the group’s members possessing that feature, according to non-summativism. More radically, on some non-summativist views, the group’s possession of the relevant feature may not even partially consist in any group members possessing that feature: it may be possible for a group to possess a virtue, vice, or character trait that none of its members possess.

There are two main kinds of argument for non-summativism about group character traits one can find in the literature. The first kind, called “divergence arguments”, appeals to cases in which group members tend to behave in a markedly different way in the group context than they would outside of it (e.g., Fricker, 2010; Lahroodi, 2007). In these examples, a group appears to display a character trait while the group members in their private lives appear not to display it, or a group appears not to display a character trait, though its members do appear to display it in their private lives. Often, what plays a key role in these examples are the group’s policies or procedures, whether formal or informal. The group has adopted policies or procedures that regulate their members’ conduct when acting as group members, and these policies or procedures lead the group members to behave differently as group members than they would as private individuals. The group tends to display (or not) a unified range of behaviors because of group values encoded in the group’s policies and procedures, but the individual group members, because they may not endorse these same values equally as private individuals, govern their private conduct differently. This might lead, for example, to racist groups composed of non-racist individuals, or the opposite.

A second kind of argument for non-summativism appeals to the idea of “distinctively collective virtues”. This kind of argument focuses on cases in which a group appears to manifest a character trait that just isn’t available as a character trait for individuals, because of differences between groups and individuals (see Byerly & Byerly, 2016). The most obvious example of a relevant difference between groups and individuals is that groups have members who may interact in the group’s activities, whereas individuals do not. As such, if there are any character traits concerned specifically with the regulation of group member interaction in group activity, these may be good candidates for distinctive group character traits that cannot be possessed by individual inquirers. Candidates for such virtues have included solidarity (Battaly, forthcoming) and excellent distribution of labor (Byerly, forthcoming).

In this section, I will consider the debate between summativist and non-summativist views as applied to the character trait of intellectual transparency. Thus, my question is: for a group to possess the trait of intellectual transparency as described in the previous section, is this always just a matter of the right combination of the group’s members possessing intellectual transparency individually (summativism), or not (non-summativism)? I will defend a non-summativist answer. In many cases, group intellectual transparency consists in more than just the right combination of intellectual transparency among group members.

Perhaps more interesting than the answer is the way I will defend this answer. The argument for non-summativism that I will offer here is a different kind of argument for non-summativism than the two kinds just noted. It is not based on the idea that individuals might behave differently with respect to intellectual transparency in the group context than outside of it, nor is it based on the idea that intellectual transparency is a distinctively collective virtue. Indeed, I wish to argue that even if intellectual transparency can be possessed by both individuals and groups (and so it is not a distinctively collective virtue), and even if the members of a group possess the virtue of intellectual transparency themselves and act in accordance with it in the group context, this is not always all there is to the group’s possessing intellectual transparency. Group intellectual transparency can and typically does require or consist in more than group members’ intellectual transparency.

The main argument I will advance for non-summativism can be stated straightforwardly as follows. For a group to possess intellectual transparency, per our hypothesis, is for the group to be disposed to attend well to the group’s perspective and to communicate this perspective well to others within and outside the group out of a motivation to promote others’ epistemic goods. Yet, for a group’s members to individually possess intellectual transparency, they do not need to attend well to the group’s perspective and communicate this well to others. Instead, they need only to attend well to their own individual perspectives and communicate these to others well out of a motivation to promote others’ epistemic goods. Moreover, group members doing the latter, I will argue, will not always or even typically suffice for the group’s having attended well to its perspective and communicated it to others well. Thus, there is something required for a group to possess intellectual transparency that is often not satisfied merely by the group members possessing intellectual transparency: namely, the group’s being attentive to and communicating well to others the group’s perspective.

While this main argument may be clear enough as far as it goes, it will prove helpful to consider in more detail the key contention that a group’s being attentive to its perspective and communicating this perspective well is often not secured merely by the group members attending individually to their own perspectives and communicating these well. To facilitate consideration of this topic, I will examine four features of groups’ perspectives and argue that attentiveness to and disclosure of these features of a group’s perspective is often not secured merely by group members’ attentiveness to and disclosure of their perspectives. Specifically, I will argue that a group’s attentiveness toward and disclosure of the group’s beliefs or acceptances, evidence, processes of inquiry, and intellectual tendencies is not always secured merely by group members’ attentiveness toward and disclosure of their own individual beliefs or acceptances, evidence, processes of inquiry, and intellectual tendencies. Nor is a group’s attentiveness toward and disclosure of these features always secured through group members’ attentiveness toward and disclosure of other features of their individual perspectives. Thus, for each of these kinds of features of group perspectives, something more is often required for the group to attend to and disclose these features well to others than for the group members to act in accordance with intellectual transparency.

### Group beliefs or acceptances

I’ll start by considering group beliefs or acceptances, which I think furnish the weakest example for purposes of defending the present argument. While they provide the weakest example, I will consider them at greater length than I consider the other cases. This is because in the process of considering them I will introduce key ideas that will be relied upon in later arguments, and also because defending the argument in this case, given its more demanding nature, requires somewhat greater care.

We can begin by noting that the summativist versus non-summativist debate recurs for most of the features of group perspectives I will examine, and is not isolated to consideration of group virtues, vices, and character traits as noted earlier. Thus, it has been debated whether for a group to believe something is just for the right combination of its members to believe it (a summativist view of group belief) or not (a non-summativist view). The trend in philosophical work on the topic has been toward non-summativism (see Lackey, 2020). For a group to have a belief is often something more than or other than for the right combination of its members to have that belief. If this is true, then for a group to attend to its beliefs and communicate these effectively to others may require more than for the members to attend to their beliefs and communicate these effectively to others. Group beliefs and group member beliefs have a different ontology. Even if all group member beliefs have been attended to and communicated, this does not imply that the group’s belief has been attended to and communicated.

Several lines of argument have been given in favor of non-summativism about group belief: I’ll mention two (cf. Gilbert, 1989; Schmitt, 1994). First, each person in a group might hold a belief about whether p or not-p; yet, the group as a whole may never have considered as a group whether p or not-p. And, it may seem in such cases that the group does not have a view on the matter. Thus, group beliefs are not always constituted merely by group member beliefs. Second, groups may adopt standards of evidence that lead members to not be willing for their own private beliefs to stand as the group’s belief. This may happen for juries, for example, in which each jury member may privately believe a defendant is guilty, but the members are not willing to give a guilty verdict as a group because they think that to do this stronger evidence is required than is needed to support their individual beliefs of the defendant’s guilt. Here again, it may seem that the group’s belief is not constituted just by the group members’ beliefs.

These kinds of arguments have led some philosophers to develop accounts of group belief that do not appeal to group member beliefs. The standard account along these lines, due to Margaret Gilbert (1989), appeals to the idea of joint acceptance. For a group to believe p is for the members to jointly accept p. Joint acceptance itself is explained in terms of the group members having common knowledge that each has openly expressed their willingness to accept p along with the other members. Thus, a group believes p when and only when the group members have expressed their willingness to accept p along with the other group members, and this willingness of the group members is commonly known within the group.

If this kind of account of group belief is correct, then it is clear that for a group to attend to and communicate its beliefs requires more than for its members to attend to and communicate their own individual beliefs. Indeed, it would appear on this account that group members’ attending to their beliefs and communicating these to others in the group or outside of it makes no contribution to the group’s attending to and communicating its beliefs. Group beliefs are just made up of different stuff than individual beliefs. For the group to attend to its beliefs and communicate these, on this account, the group needs to attend to what the group members jointly accept, and this is not secured by the members each attending to and communicating what they believe.

Now, importantly, the argument thus far does not yet get us the conclusion we are interested in—that group members attending to and communicating their perspectives well is not always enough for the group to attend well to its beliefs (if any) and communicate these well. This is because it could be that a group’s attending to and communicating well its beliefs is secured not by its members attending to and communicating well their beliefs, but instead by their attending to and communicating well other features of their perspectives besides their beliefs. Thus, for instance, perhaps the jury won’t count as having attended to and communicated its beliefs about the defendant’s guilt well because its members each attend to and communicate their beliefs about the defendant’s guilt; however, perhaps the jury will count as having attended to and communicated its beliefs about the defendant’s guilt well if the members each attend to what they accept regarding the defendant’s guilt and communicate this well.

If Gilbert’s account of group belief is correct, then I do think that if group members behave in this way regarding what they accept as group members, this gets us much closer to the group itself exhibiting intellectual transparency regarding its beliefs. However, it does not quite get us all that is required, and this is crucial. The basic point I want to make here, which will carry over to what I will say about other topics, is this: typically, it is a different thing for each member of a group to attend to their own perspective and communicate it and for someone—anyone—to attend to and communicate the group’s perspective. Yet, for a group to exhibit intellectual transparency, someone must attend to and communicate the group’s perspective. In at least some cases, no amount of individual members attending to and communicating their own perspectives will do the trick.

To see how the point applies to the case we are currently considering, it will help to notice that on Gilbert’s view, the group’s beliefs consist not in what any individual member accepts, but instead they consist in what the group members *together jointly* accept. To attend to and communicate what the group believes, someone would thus have to attend to and communicate what the group members jointly accept. But the group members attending to their own individual perspectives and communicating these well does not guarantee that anyone will have attended to and communicated well what (if anything) the group members jointly accept. If the group members themselves have each accepted certain views as members of the group, then they will each individually attend to and communicate this—but this is not by itself enough for anyone to have attended to and communicated what (if anything) the group members together jointly accept.

Consider how this might play out in the jury case. The jury goes away and deliberates. Each member has their own beliefs about the guilt of the defendant. Let’s suppose that each of them attends well to their own perspective about this, and is highly confident about their own position, reveals this position to the group, and—given their confidence—each accepts their own view of the matter to be the group’s view. The trouble is, they don’t listen to each other’s views. Each just assumes that the others also accept the same view as the group’s view. But they are wrong: the members of the jury take different views about the defendant’s guilt. The time comes for a verdict to be given. Instead of a spokesperson acting on behalf of the jury, each member of the jury reveals their own perspective, doing a stellar job of representing their individual perspectives faithfully. One says they have accepted as the group’s view that the defendant is guilty, and so has everyone else. The next says they have accepted as the group’s view that the defendant is not guilty, and so has everyone else. And so on down the line.

Now, there are two questions for us to consider about this case. First, did each member attend well to their own perspective and communicate it well to others? Here I think the answer is ‘yes’. It is true that part of the case as described is that each juror’s communication of their perspective was not taken up well by their fellow jurors. But this doesn’t imply that the jurors didn’t communicate their perspectives well—it’s just that the fellow jurors were obstinate in their inattentiveness to one another. Individually, each juror behaved in accordance with intellectual transparency. The second question is this: did the group attend well to its beliefs and communicate these well to others? Here I think the answer is ‘no’. Nobody, certainly none of the jurors, attended well to what (if anything) the jurors jointly accepted regarding the defendant’s guilt. The jurors each attended to their own perspectives, including what they each accepted as being the group’s view, but none of them attended well to what (if anything) the jurors together jointly accepted. Instead, they overlooked what their fellow jurors were communicating and assumed their own views were shared by their fellow jurors.

Thus, it would appear that in this case, we have a group whose members exhibit intellectual transparency, while the group does not. The group members attend to and communicate well what they each believe and accept, but nobody attends to and communicates well what the group believes. Something more is required here for the group to attend to and communicate its beliefs, given Gilbert’s account of group belief, than for the members of the group to each attend to and communicate their individual perspectives.

In fact, the argument given here will work not only on Gilbert’s account of group belief, but on many other accounts of group belief, including those that are significantly more reductionist than Gilbert’s, and even on simple summativist accounts of group belief. I will illustrate this briefly by showing how the argument just given can be adapted to support the same conclusion using Jennifer Lackey’s recent account of group belief and a simple summativist account of group belief.

Jennifer Lackey (2020) has recently brought forward an important challenge to Gilbert-type accounts of group belief, and has proposed a more reductionist account of group belief in their place. Lackey is concerned that Gilbert-type accounts preclude the possibility of group lies. This is because for a group to lie they must communicate a view that they do not believe with the aim that others will take up this view. The problem is that on Gilbert-type accounts, by communicating in this way, the group will be jointly accepting the view communicated—and so will count as believing it, rather than not believing it. Lackey, like some others (e.g., Wray, 2001), proposes that what Gilbert provides an account of is group acceptance, rather than group belief.

Because of this problem, Lackey proposes that group beliefs must not float free of group member beliefs, but must depend much more closely on the latter than on Gilbert’s account. Yet, group belief is not just a matter of group member belief, because Lackey thinks that when group members hold the same belief on the basis of different and conflicting evidence, it is less plausible that the group itself holds the belief, given its unstable evidential foundation. This evidential instability would make such a belief impossible to coherently evaluate epistemically—but beliefs must be epistemically evaluable. Thus, for Lackey, a group believes a claim p when a significant percentage of its operative members believe p and the evidential bases of their individual beliefs in p are not “substantively incoherent” (pp. 49–50).

What I want to point out here is that on this view, the present argument for thinking that a group’s transparency about its beliefs can come apart from group member intellectual transparency will still go through. Again, imagine the jury members each attend to and communicate well their beliefs about the defendant’s guilt. They also attend to and communicate their evidence for these beliefs. But, again, the jurors don’t listen well to what their fellow jurors are communicating about this matter. They just assume that the others hold the same views they do, and even that they hold these same views on the basis of the same evidence they do. When it comes time for the verdict, they behave as before. Each communicates well their own beliefs about the defendant, as well as their evidence for these beliefs.

The trouble here, as before, is that while each juror plausibly exhibits intellectual transparency, the group does not exhibit intellectual transparency with regard to its beliefs. This is because there is no one who on behalf of the jury attends well to what is believed by multiple jurors and on what basis these jurors hold the beliefs they do, nor anyone who communicates this on their behalf. Rather, each juror individually attends well to what they believe and on what basis they believe it, and attends poorly to what the other jurors believe and on what basis they believe it.

In fact, this same argumentative strategy may succeed even on a simple summativist view that proposes that a group believes a claim just in case enough of its members believe it. Again, we might imagine that in the jury case the jurors each attend well to their own individual conflicting beliefs and communicate these well. This again will not by itself imply that anyone has attended well to what multiple group members believe and has communicated this well on behalf of the group. Yet it would seem to be the latter which would be required for someone to have attended well to what the group believes on this summativist view and to have communicated this well on behalf of the group. Thus, even on this simple summativist view, we may question whether group member intellectual transparency is sufficient for group intellectual transparency regarding group beliefs.

Of course, there are cases where it is less challenging for an audience to discern what a group’s belief is on both Lackey’s view and on simple summativist views than it is in the cases just described. We might imagine cases where there is much more agreement among the jurors in their beliefs and evidential bases, or even unanimity among them, for instance. Here we may wonder whether it is more plausible that the group qualifies as intellectually transparent simply on account of its group members’ intellectual transparency.^3^

In response, it is important to bear in mind, first, that it is no part of the view defended here that groups can never be intellectually transparent merely on account of group members’ intellectual transparency. In general, non-summativism about group intellectual character does not claim that group intellectual character traits are never constituted merely by group member intellectual character traits. While some non-summativists may push their view to this extreme, it is not required by the view itself, and some non-summativists have been clear that in their view groups can sometimes possess intellectual character traits simply in virtue of their members possessing these individually (e.g., Fricker, 2010; Jones, 2007). Perhaps the most compelling kind of case where this conclusion should be accepted is the special case in which one particular member of a group serves as the only operative member of the group, and where that person’s intellectual features stand as the group’s.

Yet, second, I also think that it would be premature to concede that in the modified jury cases in which there is more agreement among jurors we should conclude that the jury is intellectually transparent about its beliefs just because its beliefs—given the relevant views about group beliefs—are easily enough discernible to its audience. In support of this contention, there are two points to bear in mind. First, we must recall that our question is whether in such cases the group has attended well to its beliefs—which are constituted by features of multiple members of the group—and has communicated these well to others. While it may be true that others have been able to straightforwardly discern what the group’s beliefs are in this case, given the evident agreement among jurors, we may still be inclined to think that the group itself did not attend well to what its beliefs were, since no one attended to the relevant features of multiple group members on behalf of the group. Thus, we might think that while the group’s beliefs are clear enough to its audience and in that sense the group’s beliefs are “transparent”, still the group itself did not display intellectually transparent behavior in the sense we are interested in. If their group beliefs are transparent to their audience, they don’t deserve credit for making these so in the way that an intellectually transparent group would deserve such credit. Second, it should be noted that our concern is with intellectual transparency as a character trait—not just as a state. Even if we grant that in the modified jury cases the group displays intellectually transparent behavior, this does not imply that the group is inclined to display such behavior across contexts. Specifically, it does not imply that the group is inclined to display this behavior in contexts in which there is more disagreement among jurors. Thus, while we might grant that the group displays intellectually transparent behavior in this one case because there is adequate agreement among jurors, this is an accident of the particular case, and is not reflective of the group possessing the character trait of intellectual transparency—despite the fact that the group’s members may themselves individually possess this trait. The accidentality here is again indicative of the group not possessing the virtue of intellectual transparency.

Let me bring these threads together into a conclusion. The strongest view that would seem defensible in light of the foregoing arguments would be that in any case in which a group’s beliefs are not constituted in all situations by the intellectual features of just one of its members, the group will not qualify as intellectually transparent regarding its beliefs merely on account of its group members being intellectually transparent. I think the foregoing arguments provide support of this strong conclusion. But, I don’t want to insist on it. A weaker conclusion would be that, as group member beliefs become constituted across contexts by more complex and conflicting combinations of features of multiple members, it is increasingly plausible that group intellectual transparency involves more than group members being intellectually transparent.^4^ On either of these views, there will be cases in which a group’s intellectual transparency about its beliefs is not just a matter of the group’s members being intellectually transparent. And so non-summativism about intellectual transparency will be supported on the basis of considerations pertaining to intellectual transparency about group beliefs.

The arguments of this section point toward a constructive observation about what may be involved in group intellectual transparency in cases other than the special case where a group’s intellectual features consist in just the features of one particular member. Namely, in such cases, procedures may need to be set in place to ensure that someone attends well to the relevant features of multiple group members and communicates these well on behalf of the group. This might be secured by the group members simply listening well to each other and appropriately updating their views about the group in light of what they learn from each other, in addition to their being intellectually transparent individually. Or, it might be secured by an individual or subgroup within the group being tasked specifically to perform this integrative and communicative function on behalf of the group. Or, more speculatively, this function might even be discharged by a non-member on behalf of the group, in much the way that Lackey (2020) has suggested spokespeople may testify on behalf of a group. Interestingly, in any of these cases, what we have learned is that group intellectual transparency requires not just that group members reveal their perspectives well, but that someone listens well to what is revealed by group members. To borrow some of my earlier terminology, it requires not just the exhibition of virtues of intellectual dependability, but virtues of intellectual dependence—i.e., virtues that distinctively facilitate learning from others.

### Group evidence

I remarked at the outset of the previous section that in my view the case of group beliefs or acceptances furnishes the weakest example of a case where group member intellectual transparency can fail to secure by itself group intellectual transparency with respect to a particular feature of a group’s perspective. What makes this a relatively weak example is that, depending on which view of group belief we adopt, group members’ intellectual transparency by itself can and perhaps relatively frequently does make a group’s beliefs fairly transparent to their audience, even if the group isn’t itself displaying intellectual transparency. A group’s beliefs, put otherwise, can be relatively easy to discern on the basis of transparent revelation of the features of group members. But this same relationship does not hold as strongly for other features of a group’s perspective. When it comes to other features of a group’s perspective, it may remain more difficult for the group’s audience to identify these features on the basis of group member intellectual transparency alone. It is somewhat more compelling that group intellectual transparency regarding the relevant features is lacking in these cases, and the absence of group intellectual transparency also has a higher cost for the group’s audience.

We’ll begin in this section by considering the case of group evidence. Part of a group’s attending well to its perspective and communicating this perspective well to others is for the group to attend well to and communicate well the group’s evidence. Now, as in the case of group beliefs, here again, philosophers have differed regarding how they understand group evidence. Some have defended views which suggest that group evidence can come apart quite strongly from group member evidence, in a way similar to that proposed by Gilbert’s joint commitment account of group belief. For instance, on Schmitt’s view, *r* counts as part of a group G’s evidence just in case “all members of G would properly express openly a willingness to accept *r*” as part of G’s evidence (1994, p. 265). Alternatively, others have defended more reductionist views which suggest that a group’s evidence consist just in the evidence of group members. On Lackey’s (2020) view, for instance, the evidence of each group member contributes to the group’s evidence, whether that group member is an operative group member or not.

Now, again, I think the same kind of argument given in the previous section can be applied to show that regardless of which of these kinds of views about group evidence is correct, a group’s being intellectually transparent regarding its evidence is not always secured merely by the group members all being intellectually transparent individually. The group members can each attend to and communicate their own evidence (and whatever other aspects of their individual perspectives we like) without anyone attending well to and communicating what multiple members would properly express a willingness to accept as the group’s evidence, or without anyone attending well to and communicating the evidence possessed by multiple group members. Again, this is simply because the group members’ individually attending to their own perspectives does not imply that there is anyone who attends well to the perspectives of multiple group members—and yet it is the latter that is crucially required for the group to be intellectually transparent regarding its evidence, at least where the group’s evidence does not consist in the evidence of one member alone.

I’ve suggested that a group’s failure to be intellectually transparent regarding its evidence may be more costly for the group’s audience than the group’s failure to be intellectually transparent regarding its beliefs. Here’s why. Attending well to and communicating well one’s evidence is not just a matter of off-loading the whole of one’s evidence onto others. It involves sifting through one’s evidence, selecting what is relevant, considering potential tensions within one’s evidence or sources of support within it, and communicating those aspects of one’s evidence that might enhance another’s epistemic position. Remember: intellectual transparency is motivated by a concern to promote others’ epistemic well-being. The thing is, this work of sifting through one’s evidence and searching for potential tensions and sources of support within it is significant work. This is true at the level of a single individual, and can be even moreso at the level of a group whose members individually have quite different evidence. To attend well to and to communicate well the group’s evidence in such a case—particularly if Lackey’s approach to group evidence is on track—can be quite demanding. If nobody does this work on behalf of a group, and instead the group members individually each do this sort of work only with regard to their own individual perspectives, this can put the group’s audience in a tough position with respect to discerning what the group’s evidence is. The audience may need to do the sifting and searching work themselves. And, it may even be that individual members have left out bits of their evidence that seemed unnecessary to communicate considering their perspective in isolation, but are vital when considering the evidence of multiple members of the group. Thus, a group’s failure to attend well to its evidence and to communicate it well to others can put those others in a position where it is quite difficult for those others to discern what the group’s evidence is.

Constructively, the lesson here is similar to the one in the previous section. For a group to exhibit intellectual transparency with respect to the group’s evidence, procedures typically need to be put in place to ensure that someone attends well to and communicates the evidence of multiple group members (on an approach like Lackey’s) or what multiple group members would properly accept as the group’s evidence (on an approach like Schmitt’s) on behalf of the group. Here the “attending well to” and “communicating well” involves not just exhibiting receptivity to what multiple group members reveal about their own perspectives, but it also requires the activities of sifting and searching mentioned above. The fact that group members individually engage in such activities regarding their own perspectives does not guarantee that anyone engages in such activities regarding multiple group members’ perspectives. For the group to be transparent regarding its evidence, someone needs to attend well to the group’s evidence and communicate this well to others on behalf of the group—and this will often require a variety of additional features beyond the group members being individually intellectually transparent.

### Group processes

The same line of argument can be applied to group processes. Interestingly, the summativist versus non-summativist debate does not seem to have attended thus far to group versus individual processes. I do not know of suggestions in the literature that for a group to engage in an epistemically significant process P is just for the right combination of its members to engage in P. This may be for good reason. It does appear to be straightforwardly the case that epistemically significant processes can occur at the group level where these processes are not just a matter of the group members engaging in the same process individually.

One example of this is information cascades (Bikhchandani et al., 1992). In these cases, individual members in a group update their credences in light of their fellow group members’ credences without taking into account that the latter may have themselves been updated in light of other fellow group member credences. For instance, all but one member of a group may have private information that favors p over not-p, with member X being the only member whose information favors not-p over p. Yet, when X’s fellow group member X + 1 notices that X favors not-p, X + 1 updates their credence to reflect this, ending up themselves favoring not-p. And then group member X + 2 updates their credence to reflect that both X and X + 1 favor not-p. And so on, until the entire group favors not-p despite the private information of almost all members favoring p over not-p. Here a distinctive and epistemically significant group-level process has occurred—an information cascade—but this process is not one that occurs in the epistemic activity of any one individual.

A similar thing can happen in the case of conformity bias (Asch, 1951). This is a bias in which group members publicly espouse the view they perceive to be the dominant view within a group, even if they privately disagree with this view. Their public conformity to the dominant view can mislead their fellow group members to think that support for the dominant view is stronger than it in fact is, and then the latter too may succumb to conformity bias, publicly espousing this view, and so on, until the view is strongly supported by all members. Again, this process of mutually-reinforcing conformity is not something that takes place wholly within the epistemic activity of any single group member, but rather is an epistemically significant group-level process.

Now the point of highlighting these kinds of processes here is the following. Group members can attend well to their own perspectives, including any epistemically significant processes that have contributed to these, and can reveal these well to others without anyone having attended well to or communicated well to others that a group has undergone one of these kinds of epistemic processes. For instance, in the case of an information cascade, group members may individually be vividly aware of the fact that they adjusted their own credence in light of certain other group members’ credences, without taking into account whether these group members might have done the same, and they may communicate this well to others within or outside the group. But unless and until someone attends to the fact that multiple group members have engaged in this sort of activity and pieces things together, no one will have attended on behalf of the group to the epistemically significant fact that the group has undergone a process of an information cascade, nor have communicated this fact well on behalf of the group. Yet, whether the group has undergone such a process is an important aspect of the group’s perspective, and one we might hope an intellectually transparent group would be sensitive to. And similar points could be made about the process of mutually reinforcing conformity.

Here again, what stands out as significant is that for a group to be intellectually transparent regarding its epistemic processes may require that someone attends to and communicates well about these kinds of group-level processes, and this may not be secured merely by the group members each attending to and communicating their own perspectives, including those epistemic processes that partly constitute them. For a group to be intellectually transparent, processes may need to be put in place to ensure that someone attends to such group-level epistemic processes and communicates these well to others on behalf of the group. The absence of group intellectual transparency regarding such processes puts audiences in the position of having to themselves piece together whether such group processes have occurred. I would suggest that this work of piecing together lies between that required of reconstructing the evidence possessed by groups (Sect. 2.2) and reconstructing the beliefs of groups Sect. 2.1 in terms of its level of difficulty.

### Group Tendencies

Finally, we turn to groups’ intellectual tendencies. Here I mean to include the ways in which a group tends to conduct its inquiries, including any relevant intellectual character features. Sometimes, it is helpful to us to know about an individual or a group’s tendencies of this sort, so that we can better appreciate their perspective. It might help us to appreciate another individual or group’s perspective to know whether they formed it open-mindedly or not, or with intellectual humility or not, for example. In the case of groups in particular, it may even help us to know whether the group was intellectually transparent in the formation of its views. Our question here is this: for a group to attend well to its intellectual tendencies and communicate these well to others, is it always enough that the group’s members individually act in accordance with intellectual transparency? Again, I advocate a negative answer.

As with group beliefs and group evidence, both summativist and non-summativist views of group tendencies, including group character traits, have been defended. This paper is one defence of a non-summativist position, and as highlighted at the beginning of this section, there have been other defences of non-summativism about group character traits. If non-summativism about group character traits is correct, then there will be cases in which a group fails to attend well and to communicate well its character trait T but where the group’s members do all attend well and communicate well whether they individually have T. Yet, our question here is whether the members’ attending well to and communicating well the full gamut of their individual perspectives might be enough to secure the group’s attending well to and communicating its intellectual tendencies.

One route to defending a negative answer draws on the work of the previous three sub-sections. If the arguments of those sub-sections are sound, then for a group to attend well to whether it is intellectually transparent and to communicate this well to others will not always be achieved merely by the group members each attending well to their perspectives and communicating these well to others. This is because part of what is involved in a group’s attending well to whether it is intellectually transparent is a group attending well to whether it attends well to its beliefs, evidence, and processes; yet, this is not typically secured merely by the group members attending well to their own individual perspectives. The group’s beliefs, evidence, and processes are often constituted by features possessed across multiple group members. The fact that group members attend to their own individual perspectives does not imply that anyone will have attended to these kinds of features.

A broader argument, paralleling those in the previous sections, can also be given for thinking that a group’s attentiveness to and communication of other intellectual tendencies is not always just a matter of group members’ intellectual transparency. In any case in which a group’s exhibition of an intellectual tendency I is a matter of features of multiple members of the group and not just features of one member of the group, for the group to attend well to whether it has exhibited I someone will need to have attended to features of multiple members of the group on the group’s behalf. Yet, attentiveness to features of multiple members of the group is not secured, or not typically secured, merely by the members of the group individually being attentive to their own features. And, for the group members individually to be intellectually transparent only requires that they each attend well to and communicate certain of their own features—namely, their perspectives.

As in the case of group beliefs or acceptances, group evidence, and group processes, it is again plausible here that at least in many cases a group’s possessing and exercising an intellectual tendency is a matter of features possessed by multiple group members and not just a single group member. Fricker (2010), for instance, proposes that institutional virtues consist in the institution’s members jointly committing to virtuous ends for the right reason, and behaving accordingly. On this kind of view, whether a group possesses an intellectual character trait is a matter of whether multiple members have certain joint commitments—similarly to Gilbert’s account of group beliefs discussed in Sect. 2.1. In fact, even simple summativist views of group intellectual character traits, according to which a group’s possession of a trait T is just a matter of the group members possessing T, maintain that a group’s possession of an intellectual character trait depends on features of multiple group members. Thus, on these views, just as on less reductionist, non-summativist views, for a group to attend well to whether it possesses an intellectual tendency and to communicate this well, someone will have to attend well to features of multiple group members and will have to communicate about these features on behalf of the group. Yet this is not typically secured merely by the group members individually being attentive to and communicating well about their own individual tendencies.

The absence of group intellectual transparency regarding the group’s intellectual tendencies can make it very difficult for the group’s audience to discern the group’s intellectual tendencies. Intellectual tendencies like open-mindedness, intellectual humility, or intellectual transparency are often fairly complex features. To piece together whether a group exemplifies them on the basis of information relating only to the individual perspectives of group members can be a complex task. I would suggest that its level of difficulty may rival that of piecing together the group’s evidence on the basis of knowledge of only the individual group members’ perspectives. As in the cases of group beliefs, evidence, and processes, here again it would seem that in order to secure a group’s intellectual transparency, what is often needed is for processes to be put in place that ensure that someone attends well to features of multiple group members and communicates these well on behalf of the group. The relevant features here would seem to include how the group members tend to contribute to the conduct of inquiry in the group context.

## Conclusion

This paper has identified a new way of defending non-summativism about group intellectual character—particularly, about the intellectual character trait of intellectual transparency. A group’s being intellectually transparent is not always just a matter of the group members being intellectually transparent, because group intellectual transparency typically requires that someone attends well to various features of multiple group members and communicates these well on behalf of the group, but the intellectual transparency of group members individually does not usually secure this. For a group to be intellectually transparent, procedures may need to be put in place so that someone attends well to and communicates well about, at least, the beliefs, acceptances, evidence, processes, and tendencies of multiple group members on behalf of the group. This plausibly requires that someone exhibits virtues of intellectual dependence in learning from group members about their perspectives, as well as virtues of independence in evaluating the way in which the evidence, processes, and tendencies of multiple group members interact with each other. Group intellectual transparency is a complicated affair not secured merely by the intellectual transparency of individual group members.

Interestingly, this argument continues to have purchase even if simple summativist views of group beliefs, evidence, or other group intellectual tendencies are correct. Even if a group’s beliefs consist merely in the beliefs of multiple members, a group’s evidence consists merely in the evidence possessed by multiple members, and a group’s other intellectual tendencies consist merely in the intellectual tendencies of multiple members, still for the group to be intellectually transparent requires more than the group members individually being intellectually transparent. This is just because group intellectual transparency requires that someone attends to and communicates well about the features of multiple group members, but this is not typically secured by the intellectual transparency of individual group members.

Moreover, the argument for non-summativism provided here does not rely on the idea that intellectual transparency is a distinctively collective virtue, nor on the idea that group members might behave in accordance with intellectual transparency in the group context but not elsewhere, or vice versa. Thus, the argument provided here identifies a novel pathway to defending non-summativism about group intellectual character. Aside from identifying this novel argument for non-summativism, the results of the paper are important because they highlight some of the key features that may be required for group intellectual transparency beyond group members’ individual possession of intellectual transparency.

While the arguments of the paper focus on intellectual transparency in particular, one might anticipate how they could be expanded to defend non-summativist views of other intellectual virtues.^5^ The main features of group intellectual transparency that motivate non-summativism here are that it requires the group to be attentive to its own perspective and to communicate this perspective well to others. Other intellectual virtues with similar requirements may also yield to a non-summative analysis for similar reasons. For instance, virtues such as intellectual humility (Whitcomb et al., 2015) or intellectual vigilance (Roberts & West, 2015) also centrally involve the possessor’s concern for features of their own perspective. As long as the relevant features at the group level can involve features of multiple group members in the way that the features of group perspectives examined in this paper do, it would appear that an argument paralleling that given in this paper could be applied to these traits as well.

The arguments of this paper are designed to defend non-summativism as that view has typically been defined. However, some readers may still find that there is a reductive feel to the proposals offered here which may seem to be at variance with the spirit of non-summativism or particular non-summativist views.^6^ After all, the primary constructive suggestion of the paper is that for groups to possess intellectual transparency, procedures need to be put in place so that some*one* attends well to the group’s perspective and communicates this well on behalf of the group. So, it would seem that the arguments of the paper are compatible with the suggestion that a group’s possession of intellectual transparency is ultimately reducible to the features possessed by group members or representatives, even if it is not reducible to group members’ or representatives’ intellectual transparency alone. I think this is correct. That is, I think that the arguments of this paper are compatible with the idea that group intellectual transparency is reducible to the features of group members writ broadly. I am not sure that advocates of non-summativism should aim for something other than this. More importantly, the non-summativist idea that group possession of a virtue is not always secured merely through the right combination of group members possessing that virtue, and may require specific additional features even if those are features of group members or group representatives, is itself an interesting and important result for group epistemology. This paper defends this result with respect to intellectual transparency in particular, and argues that the result may be secured even while giving several concessions to summativist views.

## Footnotes

1 Indeed, some of the worries these philosophers have expressed regarding the value of transparency can clearly be seen not to pose a threat to the value of intellectual transparency as understood here, once we recognize the different ways the language of transparency is being used. Nguyen, for instance, is critical of requiring experts to reveal their reasons to the public, since doing so is not always possible and may lead experts to not use all the evidence available to them in making decisions. But intellectual transparency as understood here doesn’t require its possessor to reveal their perspective to others when doing so isn’t possible—it only involves a tendency to do this when doing so *would* advance others’ epistemic goods.

2 It might seem more economical to just use the term “character trait” here and not to mention virtues and vices separately. However, I use all three terms in order to accommodate views which propose that there are epistemic virtues or vices that are not character traits. On certain “reliabilist” approaches to virtue epistemology, this is true. See (Battaly, 2019) for discussion. Kallestrup (2020) considers the summativism vs non-summativism debate as applied to reliabilist epistemic virtues.

3 Thanks to an anonymous reviewer for encouraging me to consider such cases.

4 This weaker conclusion itself admits of stronger and weaker readings. A stronger version would contend that as the potential increases for group beliefs to be constituted by more complex and conflicting combinations of group member features, a group’s mere possession of intellectual transparency regarding its beliefs comes to require more than group member intellectual transparency. A weaker reading would contend that as the potential increases for group beliefs to be constituted by more complex and conflicting combinations of group member features, more than group member intellectual transparency becomes relevant to the extent to which the group is intellectually transparent regarding its beliefs. The latter view may allow that groups’ mere possession of intellectual transparency does not require more than group member transparency. Thanks to an anonymous referee for pointing out this possibility to me. For ease of presentation I will continue to talk about group possession of intellectual transparency sometimes requiring more than group member intellectual transparency, but readers inclined toward this weaker reading may wish to consider how appropriately modifying this language would affect the arguments that follow in both this section and the following subsections.

5 Thanks to an anonymous reviewer for encouraging me to address this.

6 Heather Battaly (forthcoming) makes some similar remarks about the analysis of solidarity, suggesting that if group solidarity is ultimately a matter of group member features, even if not a matter of group members’ own solidarity, this is in some tension with the spirit of non-summativism. A related concern is voiced by Fagan (2012) about the relationship been non-summativist and non-reductionist views of group belief. Thanks to an anonymous reviewer for encouraging me to address this.
